# Cardiac Rehabilitation: A Literature Review of Benefits, Challenges, and Emerging Approaches

**DOI:** 10.7759/cureus.95648

**Published:** 2025-10-29

**Authors:** Shaden AlHarbi, Saleha S Khan, Adnan A Moallem, Raoud M Allagani, Rand R Al Sari, Maram S Gubari, Bader M Al Murad, Nooreen Kazi, Wala S Fallatah, Sanha Sideeque, Muhammad Reihan

**Affiliations:** 1 College of Medicine, Batterjee Medical College, Jeddah, SAU; 2 General and Laparoscopic Surgery, Dr. Soliman Fakeeh Hospital, Jeddah, SAU; 3 Internal Medicine, Imam Abdulrahman Al-Faisal Hospital, Dammam, SAU; 4 Cardiology, Faculty of Medicine, Al-Azhar University, Damietta, EGY

**Keywords:** cardiac rehabilitation, cardiovascular disease, exercise training, future directions, medical technology integration, nutritional guidance, psychosocial support, risk modification

## Abstract

Cardiac rehabilitation (CR) plays a vital role in the management of cardiovascular diseases (CVDs), such as ischemic heart disease and heart failure, which remain major causes of illness and death worldwide. This literature review explores recent progress and future directions in CR, highlighting its importance as a comprehensive and multidisciplinary approach. Modern programs now combine exercise training, nutritional guidance, psychosocial support, and lifestyle modification to improve recovery and prevent further cardiac events. The inclusion of technology, such as mobile health apps, wearable monitors, and virtual rehabilitation programs, has made CR more accessible and effective for diverse patient groups. Despite strong evidence showing that CR reduces mortality, improves functional capacity, and enhances quality of life, participation rates remain low. Barriers often include limited availability of programs, low referral rates, and challenges linked to socioeconomic and cultural factors. Addressing these issues requires practical strategies to improve awareness, accessibility, and patient adherence. Future research should focus on developing culturally adaptable and technology-driven models of CR that reach more patients and deliver long-term health benefits.

## Introduction and background

Cardiovascular diseases (CVDs), principally ischemic heart disease and heart failure, have consistently persisted as the leading cause of death globally among people older than 50 years over the last three decades [1]. Additionally, it is estimated that the prevalence of most CVD risk factors and their associated morbidity will consistently increase over the next 30 years [2]. These facts highlight the importance of implementing innovative clinical and behavioral interventions to address the burden of such conditions and reduce the number of deaths caused by deteriorating cardiac functions.

Cardiac rehabilitation (CR) emerges as a revolutionary strategy that aims to improve cardiac functions among CVD patients by utilizing an individualized multidisciplinary program of life-long complex interventions [3,4]. These interventions include pharmacological treatments of established cardiac conditions, lifestyle modification and cardiovascular risk reduction, health education, as well as emotional and psychosocial well-being [3,5]. There is much evidence in the literature that supports and recommends CR as a method of reducing cardiovascular-associated mortality, hospital admissions, and costs, and improving quality of life when applied to the right candidates [6-8]. According to the National Institute of Health and Care Excellence (NICE) and the British Association for Cardiovascular Prevention and Rehabilitation (BACPR), candidates that may benefit from CR include patients with acute coronary syndrome or those undergoing reperfusion interventions, as well as individuals with chronic heart failure, a heart transplant supported by a ventricular assist device, an implanted cardiac defibrillator, congenital heart disease, heart valve replacements, or an established exertional angina [9-11].

The American Association of Cardiovascular and Pulmonary Rehabilitation (AACVPR) describes CR as providing comprehensive, long-term services that include education, counseling, behavioral interventions, prescriptive exercise, and modification of cardiac risk factors. Three steps are often involved in CR: inpatient, outpatient, and independent maintenance. Phase 1 usually occurs in an inpatient setting and involves light walking on the ward or using exercise equipment like a treadmill or stationary bike. Phase 2 typically comprises a multidisciplinary program under physician supervision and is conducted in an outpatient hospital setting. This phase consists of three components: classes on education, vigorous risk-factor reduction, and exercise. Comprehensive risk-factor reduction can treat psychosocial problems like stress, anxiety, depression, and alcohol consumption in addition to physical problems like smoking, hypertension, high cholesterol, diabetes, obesity, and diet. The goal of education programs is to provide patients with a better understanding of CVDs and give them the tools they need to take charge of their care and lifestyle changes. During phase 3, patients maintain their physical activity and risk-factor reduction on their own without cardiac monitoring [12,13]. Noticeably, exercise therapy plays a major role in the benefits and process of CR. However, the other elements are equally important. These elements might be essential to the etiology of the disease and the necessary treatment for secondary prevention, or they might be connected to common sequelae or comorbidities. For the best interest of the patient, their assessment and care are justified [13,14].

Although recommended by almost all recent clinical guidelines, CR has shown poor application and completion rates globally [7]. Many factors may contribute to this problem, including patient barriers such as gender bias and racial, socioeconomic, and language barriers, as well as poor physical health. Systemic barriers like transportation to centers, costs, and a lack of awareness among healthcare practitioners also play a role [11,12,14]. It is worth mentioning that in the Middle East and Arab countries, CVDs are very common; however, CR strategies are quite limited in these regions. Many studies have revealed a low rate of participation in CR programs because of various factors, including a lack of public awareness, restricted access to CR centers, and a lack of experts with necessary training in this field [15]. Less than 15% of eligible patients in many Arab countries, for example, are referred to or complete CR, according to research, but global rates of over 30% in certain high-income nations are reported [16]. Furthermore, this study reports that Saudi Arabia had a substantially lower rate of cardiac patients using CR services than the rest of the world, with only around 2% of eligible patients using these services [16].

Methodology 

A literature review was conducted to identify previous studies on CR, focusing on its benefits, challenges, and emerging approaches. A comprehensive search was carried out using major databases, including PubMed, Google Scholar, and Web of Science. Relevant keywords and MeSH terms such as "Cardiac Rehabilitation" [MeSH], "Exercise Therapy" [MeSH], and "Patient Compliance" [MeSH], along with terms like "tele-rehabilitation", "barriers", and "program adherence", were used to identify potential studies. A total of 112 articles were initially retrieved. The retrieved articles were screened according to predetermined inclusion and exclusion criteria to ensure alignment with the objectives of the review. Studies were included if they focused on adult patients in CR programs, reported on benefits, barriers, or innovative strategies, were published between 2010 and December 2024, and were written in English and published in peer-reviewed journals. Studies were excluded if they involved non-human subjects, were conference abstracts or editorials, were written in languages other than English, or lacked sufficient data on CR outcomes or adherence.

After careful evaluation, 78 studies met the criteria and were included in the review. The selected studies represented various research methodologies, including randomized controlled trials, observational studies, systematic reviews, and qualitative research. Only studies from reputable peer-reviewed journals were considered to ensure reliability and credibility. Final inclusion decisions were made by consensus among all authors, focusing on studies that provided meaningful insights into the benefits, barriers, and innovations in CR.

## Review

Current improvements in CR

Exercise Training

**Figure 1:**
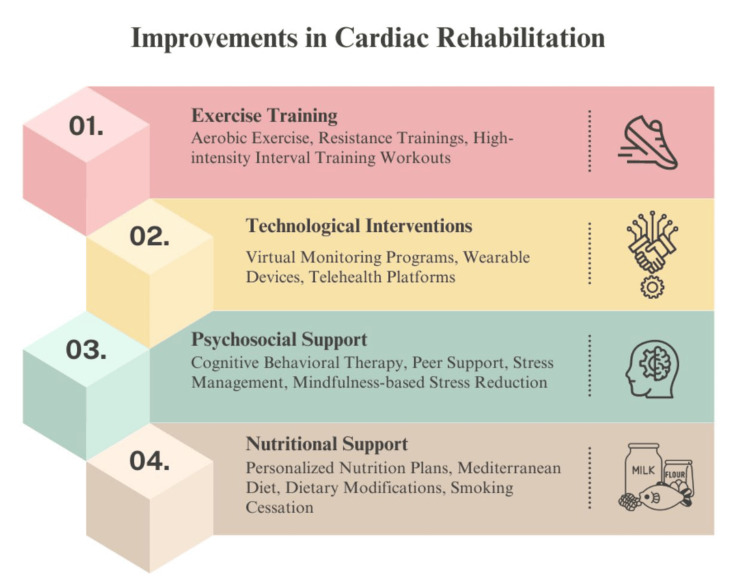
Multidisciplinary strategy for improvements in cardiac rehabilitation Image credits: Saleha S. Khan

An integral part of CR is physical exercise, as it enhances cardiovascular function, blood flow, and facilitates blood vessel dilatation. Consequently, it contributes to lowering blood pressure, increasing one's capacity for exercise, reducing mortality rates, decreasing hospital readmissions, and enhancing overall quality of life [17,18]. Furthermore, regular exercise accelerates the body's metabolism, hence reducing cardiovascular risk factors such as hypertension, diabetes, and dyslipidemia, while also providing profound benefits for mental health by alleviating psychological stress [19]. It has been found that individuals who engage in combination training (aerobic and resistance training) have a 40-46% reduction in the risk of CVD mortality and all-cause mortality, in comparison to individuals who do not participate in physical activity [20]. Physical training is a part of the recent improvement in CR among many others (Figure 1).

Aerobic training is the mainstay of exercise training [21]. Some examples of aerobic activities include cycling, running, and walking. Regular exercise has been found to enhance the heart's ability to pump blood, leading to an increase in cardiac output and stroke volume. Improving cardiovascular health reduces stress on the heart, which in turn reduces blood pressure and heart rate [22]. Other benefits of aerobic exercise include helping with weight control, increasing insulin sensitivity, and improving lipid levels. Resistance exercise focuses on one or a group of muscles. This type of exercise has been shown to improve muscle strength and endurance, which improves functional ability [17,22]. Resistance training increases bone density, thus improving bone health. Additionally, resistance training plays a role in improving insulin sensitivity, reducing cholesterol levels, and aiding in weight control by increasing metabolic rate [22]. High-intensity interval training (HIIT) is a training method that consists of periods of high-intensity training interspersed with rest or low-intensity training [18,23]. In comparison with continuous training, HIIT provides a better improvement in maximum oxygen consumption (VO_2_ max), CVD risk factors, functional capacity, cardiovascular fitness, skeletal muscle, and quality of life [24]. In addition, HIIT exercise has been found to have a similar or greater effect in reducing CVD and all-cause mortality compared to moderate-intensity continuous training (MICT) [18].

An individualized exercise regimen, tailored to the patient’s medical, psychological, and social history, can enhance participation, adherence, and the overall effectiveness of rehabilitation [17]. This customization is guided by frequency, intensity, time, and type (FITT) principles, which ensure that the exercise plan aligns with the patient’s specific needs and capabilities [21,25,26]. A total of 150 minutes of moderate-intensity exercise distributed over 3-5 days per week is the recommended aerobic activity [22]. Strength training is also recommended, typically 1-3 sets of 8-12 repetitions performed at least twice weekly [20,22]. For patients suited to more advanced protocols, HIIT can be incorporated, typically involving a warm-up session, several four-minute intervals at 75-90% of peak heart rate with active recovery at about 60%, followed by a cool-down [17].

Knowing an individual's maximal exercise ability enables the physician to formulate a personalized exercise program that is effective and safe [23]. The most used exercise test is cardiopulmonary gas analysis. This test can help to determine a suitable exercise prescription for patients by providing indices such as oxygen consumption (VO_2_) reserve, heart rate reserve, ventilatory threshold, and workload [23,26].

Additionally, the cardiopulmonary exercise test can also determine any unusual cardiovascular changes, including arrhythmias, changes in blood pressure, or abnormalities in the ECG [23]. However, in patients with atrial fibrillation, heart transplant, cardiac pacemakers, chronotropic incompetence, or those receiving beta-blockade medications, obtaining a heart rate during exercise can be challenging [18]. In these cases, subjective methods (e.g., rating of perceived exertion (RPE) or talk test) can be used to determine the exercise intensity. The Borg scale of RPE allows the patient to report their perceived exertion of exercise on a scale ranging from six (no effort) to 20 (maximal effort) [27]. The talk test is another subjective method to evaluate exercise intensity. This test relies on the rapid rise of breathing beyond the lactate threshold, which leads to challenges in maintaining comfortable speaking during exercise. Therefore, it helps determine the demarcation point between moderate and vigorous exercise intensity [18]. As for measuring the intensity of resistance exercises, the one-repetition maximum test (1-RM) is used. This test determines the maximum weight an individual can successfully lift through a full range of motion in one repetition [17,23]. The recommended initial intensity for resistance training is about 40-60% of 1-RM, after which a gradual increase in resistance is recommended over time [20].

Recently, the combination of remote technologies and wearable devices has improved the monitoring of physical activity performance in patients exercising at home [28]. This includes several measures of physical activity such as duration, intensity, distance, sitting time, and steps taken. In addition, sensors can retrieve data related to blood pressure, heart rate, and ECG [28,29]. Subsequently, professionals can evaluate this data using technology applications, allowing for continuous monitoring and instant feedback between CR providers and patients [28]. Furthermore, wearable devices incorporating ECG and accelerometers, when combined with artificial intelligence (AI) algorithms and continuous data monitoring, facilitate more accurate identification and detection of an individual's physical activity. Additionally, using online platforms that are AI-powered enables remote feedback and communication between the patients and the CR providers, thus enabling consultations and modification of physical activity plans [28].

Technology Integration

Historically, patients have only had the option of in-person CR; however, the rapid pace of advancement in technology has increased the number of readily available wearable devices and smartphone applications. Therefore, the integration of digital CR platforms has been evolving steadily to enhance functional status, monitor patients' progress, facilitate adherence to CR programs, and improve quality of life for patients with CVDs. Moreover, amid the COVID-19 pandemic, there was a substantial surge in adopting virtual and hybrid CR programs by healthcare providers as a result of reduced or suspended in-person services [22,30-33].

Virtual monitoring programs employing innovative technologies have demonstrated a significant increase in adherence, as they provide well-structured, accessible, and flexible options that align with patients' needs while ensuring quality outcomes. Consequently, these programs have addressed various challenges associated with traditional in-person CR, including commuting issues, physical limitations, and multiple competing priorities [22,30-32].

Moreover, CR virtual coaching programs offer synchronous audiovisual consultation, for example, with a registered dietitian for weight management or with a behavioral therapist for smoking cessation, or an asynchronous remote model could be provided [30].

As CR evolves, hybrid models that blend in-person and virtual components are increasingly being adopted by healthcare facilities. A study compared outcomes across three delivery methods: in-person, hybrid, and virtual. In the virtual group, participants attended a maximum of two in-person visits for clinical metrics collection, while the hybrid group had at least three in-person visits, with two potentially for metrics collection. All groups demonstrated similar improvements in functional capacity. Additionally, the in-person visits fostered strong rapport between participants and staff, while the virtual sessions facilitated continuous connections and allowed for prompt resolution of participation barriers. Moreover, the study allowed patients to switch session types during the study to accommodate patients' preferences or medical recommendations, which subsequently enhanced accessibility, adherence, and mentoring; thus, integrating hybrid models into CR programs is crucial, as they deliver great benefits to patients without compromising outcomes [22]. However, despite multiple evidence supporting the effectiveness of remote monitoring and integration of tech innovations, several challenges still exist. One of these barriers is the lack of standardization among devices and data from various manufacturers. Furthermore, most data applications are being sold to third parties by app developers, which can compromise data security and safety; such distrust regarding the security of data can impact a patient’s willingness to use these applications [32].

Cultural and socioeconomic factors, including a patient's financial status, living in an area without a CR center or a single center, internet access, and technical features, are key determinants in influencing participation in virtual CR [22,34]. Indeed, numerous studies demonstrate that older adult patients are willing to incorporate wearable devices into their daily routine if the devices are lightweight, easy to operate, and more affordable [33]. Additionally, transitioning to virtual CR or hybrid programs entails adopting new delivery models that demand highly trained staff, resulting in added costs for hospitals. Moreover, issues related to insurance reimbursement and patient out-of-pocket expenses pose significant challenges for hospitals and health systems in establishing sustainable virtual CR programs [22,30]. Highlighting the significance of addressing cultural and socioeconomic factors and integration of cost-effective technologies is particularly vital for the successful implementation and sustained usage of scientific advancement. In addition, the trials conducted thus far have had limited sample sizes and brief randomized trial durations, necessitating longer-term follow-up to better understand the impact of these interventions on a broader scale and higher-risk populations. Consequently, it is not yet possible to draw firm conclusions regarding the role of digital health literacy among the patients [30-32].

The prospects of integrating more technology into CR programs hold significant promise due to advancements in precision medicine and patient-centered care [34]. By enhancing hybrid CR models, patients can select the delivery format that best aligns with their individual needs and circumstances, making participation more accessible. Ongoing assessment of patients' needs and preferences is of paramount importance for the successful development of these programs. Future research is needed to address models’ effectiveness for all populations, including women, minorities, patients with disabilities, and high-risk groups that are often underserved by current CR programs to ensure successful advancement of CR programs [22].

Psychosocial Support

Psychosocial factors, including stress, depression, anxiety, and social support, have a significant impact on the outcomes of CR. Indeed, effective stress management techniques are crucial because excessive levels of stress can exacerbate cardiovascular symptoms and prolong recovery [35]. Cognitive-behavioral therapy (CBT) has proven to be beneficial since such an approach assists in restructuring negative thought patterns into positive ones and adopting more constructive coping strategies. In cardiac populations, CBT not only reduces stress, depression, and cardiovascular risk factors, such as blood pressure and cholesterol levels, but also promotes adherence to medication, dietary modifications, and exercise regimen [36-38].

Social support is an integral determinant factor in recovery and long-term illness management. In cardiac patients, improved adherence to treatment plans, dietary recommendations, and physical exercise has been repeatedly linked to familial, peer, medical, and community support on an emotional, informational, and practical level. Peer support interventions strengthen self-management behaviors, accountability, and social connectedness [36-40].

Complementing these interpersonal forms of support, significant gains in psychological well-being have also been linked to mindfulness-based stress reduction (MBSR), which emphasizes present-moment awareness through meditation techniques. Research shows that MBSR improves emotional resilience while lowering stress, anxiety, and depression. Better adherence to self-care practices and positive impacts on physiological markers, such as inflammation and heart rate variability, have also been associated with MBSR participation [39].

A recent Cochrane systematic review and meta-analysis of 21 trials (n = 2,591) conducted in 2025 provides additional evidence supporting the use of psychological therapies in cardiac populations. Results showed that at 6-12 months of follow-up, there were substantial reduction in depression (standard mean difference (SMD) -0.36; 95% confidence interval (CI) -0.65 to -0.06; p = 0.02) and anxiety (SMD -0.57; 95% CI -0.96 to -0.18; p = 0.004), as well as improvements in mental health-related quality of life (SMD 0.63; 95% CI 0.01 to 1.26; p = 0.05). However, there were no discernible impacts on major adverse cardiovascular events, all-cause mortality, or quality of life related to physical health. These results imply that psychosocial therapies do not directly affect survival outcomes but rather improve psychological well-being, adherence, and self-management [41].

The practicality of integrating these interventions into standard clinical practice was highlighted by a noteworthy high adherence and acceptance (approximately 71%), with a low incidence of adverse events across trials [41]. Therefore, combining CBT or MBSR with structured exercise and pharmacological treatment appears particularly promising, as they address both psychological and physiological aspects of recovery. However, the long-term sustainability of these methods remains limited; the certainty of the evidence is rated as low for quality-of-life outcomes and moderate for depression and anxiety outcomes [38-41].

Motivational interviewing (MI) has also drawn interest. MI has been demonstrated to raise patient engagement, improve health-related quality of life, and improve adherence to self-care in heart failure patients. MI supports long-term behavioral change and enhances clinical outcomes, reinforcing its integration into comprehensive CR, according to a recent systematic review and meta-analysis [42].

There are opportunities as well as challenges when incorporating psychosocial interventions into routine CR programs. More research is needed to assess the combined effects of interventions such as CBT, MBSR, and peer support within multidisciplinary models of care, even though their benefits have been clearly demonstrated. Further studies are also required to elucidate the sustainability of these benefits over time, including long-term adherence and maintenance of behavior change [40].

Nutritional Guidance

In CR, incorporating dietary interventions and customized nutrition regimens is essential since it presents numerous opportunities to improve cardiovascular health and reduce the risk factors for recurrent cardiac episodes. The most recent research emphasizes how important structured nutritional counseling and education are to rehabilitation procedures. It reviews the newest evidence supporting these approaches and classifies key research gaps and recommendations for upcoming studies, providing a comprehensive overview of how nutritional strategies can develop outcomes for cardiac patients [43]. According to another recently published literature, some dietary treatments can promote cardiovascular health during CR. Personalized nutrition plans tailored to individual patient needs and health conditions have been found to enhance recovery and long-term well-being in several trials. In most cases, this approach concentrates on encouraging healthy eating habits, which are often low in saturated and trans-fats, sodium, and added sugars, but high in vegetables and fruits, whole grains, lean meats, or poultry without the skin. These changes in the diet lead to a decrease in blood pressure levels, total cholesterol levels, and body fatness, thereby reducing the chance of recurrence of cardiovascular hazards [43,44].

A Mediterranean lifestyle diet is also found to be beneficial toward reducing cardiovascular events in healthy individuals; Following a Mediterranean diet plan has been shown to have a lot of potential for CR. This dietary strategy, which is marked by an abundance of fruits, vegetables, whole grains, legumes, and unsaturated fats, has been associated with a decrease in cardiovascular risk factors like inflammation, dyslipidemia, and hypertension. Additionally, adding fish and olive oil supplies vital nutrients that promote heart health. Although more research is needed to determine the exact mechanisms underlying these advantages, the Mediterranean diet presents a viable nutritional approach for enhancing patient outcomes in CR [44,45].

Multiple encouraging results have been demonstrated from investigating nutritional advocacy and education in CR protocols. Research done together shows that clients who receive organized nutritional information are more likely to follow heart-friendly foods, hence leading to developed cardiovascular outcomes [43].

Several important research gaps in the nutritional strategies used for cardiac patients have been identified by recent literature. Nutritional plans that are more detailed and customized will be necessary because they must consider metabolomics, microbiome, genetics, and certain specific biomarkers. It is essential to have a comprehensive understanding of how these variables influence and how each person reacts to dietary interventions. Research on the most effective exercises for implementing dietary counseling in conventional CR programs ought to be the next focus. This includes the development of standardized methods and instruments that would guarantee high nutrition intervention compliance and efficacy [46-49].

Current studies on the effects of various food components, such as different types of fats, carbohydrates, and proteins, on cardiovascular health are inconsistent. More robust, long-term research is required to identify ideal diets and clarify these associations. Additionally, the diversity of existing studies is limited, with an underrepresentation of different racial, ethnic, and socioeconomic groups.

Hence, in order to better understand how nutrition strategies can fit in, future research should try to include these populations [46,47]. By doing this, future research will offer more comprehensive and effective nutritional strategies that can improve the overall care and rehabilitation outcomes for cardiac patients. However, from a broad perspective, while dietary interventions and individualized nutrition plans are being supported by contemporary data in the integration of CR processes, this research is necessary to refine these approaches to respond effectively to the different requirements of cardiac patients [47].

Risk Modification

CR has evolved into a multidisciplinary approach that emphasizes patient education, individualized exercise training, and other key aspects of overall health in cardiac patients. It has been well proven to be an effective treatment modality for heart diseases. Studies in recent years have shown that, when delivered in a timely manner, CR offers proven benefits to individuals with a variety of cardiac conditions, including ischemic heart disease, heart failure, arrhythmias, and post-cardiac surgery. As part of the program, each participant receives individualized instruction on dietary salt intake, weight-loss strategies, and lifestyle modifications such as the Mediterranean diet or the dietary approaches to stop hypertension (DASH) diet [48-50]. Following a healthy lifestyle that incorporates regular physical activity, no smoking, a balanced diet, and maintaining optimal cholesterol and blood pressure levels is associated with a substantially lower lifetime risk of heart disease and better preservation of the heart's structure and function. Thus, lifestyle counseling remains an obligatory part of CR [51].

For weight management, patients with a BMI ≥25 kg/m² have frequently been included in CR. The American Heart Association/American College of Cardiology (AHA/ACC) recommends a BMI of 18.5-24.9 kg/m²; however, few participants reach this goal by program completion. Nevertheless, overweight or obese patients are advised to reduce their body weight by 5-10% over six months. Along with lifestyle modifications, CR programs encourage adherence to guideline-based medical therapy. For instance, the AHA/ACC guidelines suggest that patients with atherosclerotic cardiovascular disease (ASCVD) be treated with high-intensity statins that lower their low-density lipoprotein cholesterol (LDL-C) by at least 50%. Desirable treatment goals for LDL-C are <100 mg/dL for lower-risk patients and <70 mg/dL for very high-risk patients, supplemented by non-statin therapy if necessary. By formally educating patients, closely following them, and always supporting them, CR helps patients comply with these pharmacologic regimens within the context of adopting a healthier lifestyle. Furthermore, comprehensive management of risk factors is central in CR. Diabetes, present in approximately 22% of CR participants and increasing steadily, is addressed through CR programs, where a targeted level of hemoglobin A1c <7% is achieved with supervised exercise, nutrition modification, and encouragement of medication compliance. Hypertension, which affects 60-70% of participants, is controlled in a similar fashion - with aerobic training, weight loss, and stress reduction - as an adjunct to pharmacotherapy, allowing patients to reach guideline-dictated targets of <140/90 mmHg for most persons and <130/80 mmHg for those with comorbid disorders. Tobacco cessation continues to be an integral priority, as 5-16% of patients still smoke; CR programs offer counseling, peer support, and pharmacological interventions to further increase long-term abstinence. Exercise training underpins all aspects of CR, yielding broad metabolic and cardiovascular benefits. It improves insulin sensitivity and glycemic control by lowering hemoglobin A1c by 0.6-0.8% in type 2 diabetes, while also reducing systolic and diastolic blood pressure by 2-7 mmHg and improving lipid profiles by decreasing triglyceride (5-25 mg/dL) and LDL-C (3-10 mg/dL) levels and raising high-density lipoprotein cholesterol (HDL-C) (2-5 mg/dL) levels. Although average weight loss over six to 12 months is modest (1.6-1.7 kg), exercise training significantly reduces liver and visceral fat, thereby addressing risk factors closely linked to type 2 diabetes and CVDs. Together, these interventions highlight CR as a comprehensive framework for secondary prevention and long-term cardiovascular health [51-53].

Future directions

Virtual Reality (VR) and AI Integration

Recent advancements in technology have had a huge positive effect on CVDs. By helping create user-friendly and immersive virtual environments that feel realistic, these innovations have had positive effects on cardiovascular health, such as improving early diagnosis and treatment, reducing mortality and healthcare costs, and enhancing patient quality of life and satisfaction [54,55].

Patients with CVDs are often faced with depression and anxiety, and that can negatively affect the effectiveness of CR [56]. CR has long been considered essential for people recovering from heart attacks or strokes. VR-based therapy for anxiety and depression provides intense stimulation of the senses and has been shown to reduce these symptoms effectively, making it a promising addition to traditional CR programs [56]. The Virtual Therapeutic Garden supports CR; the garden begins as dull and gradually becomes brighter and more vivid with each session, signaling the patient's journey toward recovery, which is focused primarily on Erickson’s therapy [55]. The success of the garden is primarily dependent on the interventionist's ability to mentally piece together the patient's anatomy from various imaging modalities [56].

Exercise-based CR is recommended after heart valve surgery and has demonstrated positive short-term improvements in the physical capacity of patients. However, receiving rehabilitation with a stressed or disabled body is a boring and exhausting battle. Software such as Microsoft Kinect (Microsoft Corp., Redmond, USA) has played an invaluable role in CR [57], as patients don’t need to wear tools related to the application or visit hospitals just for their CR. Exergaming, a VR-based aerobic device, utilizes VR to create a video game-like environment for the users. It helps to perform physical tasks by engaging with the virtual world [58].

Immersive VR therapy offers significant benefits for those with anxiety and depression symptoms during CR. VR can reduce anxiety in patients during their hospital stay, undergoing transcatheter aortic valve replacement (TAVR), by providing relaxing VR videos. VR and augmented reality (AR) have been found to increase the effectiveness of traditional physical training. For patients who are unwilling to engage in traditional activities or live in remote locations without access to in-person programs, programs with VR and AR can be a lifesaver. VR has been applied to every stage of CR. After cardiac surgery, Cacau et al. used VR to help patients with motor exercises, and they saw faster recovery times and earlier hospital discharges [56]. CR methods with VR are suggested to be more engaging and enjoyable compared to repetitive traditional exercises [58].

A lot of important work is to be noted as VR and AR technologies continue to advance. Enhancing the physical and tactile sensations will help make the virtual experiences for patients more realistic and engaging. The environments will be more helpful if they are more realistic and captivating. In this developing world, improving the portability, affordability, and accessibility of technology and software can be crucial for CR. However, the expenses and complexity of these systems may act as a barrier.

Innovative Therapies and Interventions

Recently, CR programs have embedded an approach to personalized medicine using technological interventions such as wearable devices, mobile applications, and telehealth platforms that remotely monitor patients’ vital signs, physical activity, sleep patterns, and more. This data can track patient progress and adjust treatment plans accordingly. These technologies provide real-time feedback, target setting, motivational support, and encourage patients to participate in their CR program [59]. In addition, pharmacogenomics techniques are being used in tailoring the most effective and safest medication regimen by providing a guide for medication selection and dosing, for instance, medication for hypertension and dyslipidemia [60]. Biofeedback interventions are increasingly being used in rehabilitation. Studies have shown that biofeedback can positively affect cardiac health. In one study where patients used a cycle ergometer, they received feedback in the form of cadence, allowing them to control their pace during the session. This helped stabilize cardiovascular and respiratory parameters and improved exercise tolerance. Additionally, CR programs have embraced heart rate variability biofeedback (HRVB), which is a non-invasive respiratory feedback technique that focuses on the autonomic nervous system to adjust the respiratory rate to resonance frequency, hence, improving the patient’s heart rate variability [61].

HRVB also includes psychological rehabilitation, such as mental stress. A personalized biofeedback system was designed by integrating HRVB into a cognitive module, named “FreeRasp." This system combines the advantages of wearable devices and the Internet of Things and incorporates a simple human-machine collaborative cognitive decision-making framework. Hence, the "FreeRasp" system provides real-time data collection and analysis that is used to produce personalized resonance frequency and efficacious HRVB and enhance training efficiency [62].

Moreover, yoga plays a great role in modifying risk factors affecting heart diseases. Since yoga reduces stress and depression levels, it has a positive effect on heart rate, respiratory rate, oxygen consumption, and other psychological effects. Studies have shown that patients participate in yoga because it has easy practices that can be performed at home with practical low cost [63]. Despite yoga's potential effectiveness in CR, more randomized controlled trials with larger sample sizes and diversity are required to evaluate its efficacy [64].

Acupuncture has been approved to improve hypertension and psychological stress-induced cardiovascular activity, thus preventing CVDs [65]. According to a study, the application of acupuncture in CR can regulate heart energy metabolism, enhance the effectiveness of exercise ventilation, promote fatty acid oxidation, and improve depression and anxiety symptoms. However, the mechanism of acupuncture remains unclear; thus, deeper experimental studies with larger sample sizes are needed to assess the efficacy of acupuncture on cardiac patients [66].

Access and Equity

Since the onset of the 21st century, CR has seen a marked advancement in research, innovation, and its diverse approach to patient care. AI, telehealth solutions, and innovative devices have enhanced the convenience of rehabilitation [67,68]. As a result of these advancements, CR has become a worldwide issue, leading nations to create policies and institutions like the Global Cardiac Rehabilitation Registry (GCCR) to promote collaboration [69]. The development of this program and its effects on patient outcomes are well-documented by research. Nonetheless, disparities, obstacles, and inequities still exist despite the obvious advantages [70].

Disparities in CR participation according to risk ratios, demographic, socioeconomic, and geographic factors, as well as the accessibility of curative and preventative medicines, have been repeatedly brought to light by research. Research shows that these differences are largely caused by low referral rates from healthcare providers. For instance, numerous literature evaluations have noted that vulnerable groups and minority populations, such as Black, Hispanic, and Native African American people, frequently experience poorer referral and completion rates than their White counterparts. Regarding gender equality, one meta-analysis of 19 observational studies documented that 32% women were referred less than men despite the eligibility criteria [71]. Socioeconomic position, lack of insurance coverage, transportation challenges, cultural views, and a lack of knowledge about CR advantages are some of the factors that contribute to these differences [72-74]. 

In order to enhance access to CR and address these inequities, comprehensive initiatives are necessary. Ensuring culturally sensitive care and identifying eligible patients through comprehensive screening methods are important initial steps. Rural and isolated communities can have higher participation rates by overcoming geographic barriers using telehealth and home-based CR models [72-75]. Financial incentives such as reimbursement reforms and subsidizing transportation costs can alleviate financial burdens associated with CR participation. Furthermore, community partnerships, peer support programs, and patient education initiatives tailored to diverse populations can improve awareness and engagement. Hence, referral strategies, automotive referrals, home-based CR, equitable access to technology, and workforce diversity could improve access, reduce barriers, and enhance equity in CR services [72-75].

Promoting equity in CR services is mostly dependent on policy actions. It is possible to standardize access and guarantee affordability for all eligible patients by promoting mandated referral guidelines and insurance coverage for CR treatments [11]. In order to improve continuity of treatment, healthcare reforms should give priority to financing CR initiatives in underprivileged areas and incorporate CR into frameworks for managing chronic diseases. To guide focused interventions and accountability measures, quality metrics and monitoring systems that track differences in CR utilization across demographic groups can be established [30].

Healthcare systems should strive toward attaining equitable delivery of CR services by putting inclusive methods into practice, such as increasing awareness, enhancing cultural competency, utilizing technology, pushing for policy reforms, and encouraging collaboration.

## Conclusions

CR remains a cornerstone in the management of CVDs, offering proven benefits in reducing mortality and improving quality of life. This review uniquely integrates evidence from recent advancements across multidisciplinary domains, combining traditional CR principles with innovative approaches such as AI, VR, biofeedback, and personalized medicine. Unlike earlier reviews that focused mainly on exercise or psychosocial factors, this study presents a comprehensive perspective linking physiological, technological, and behavioral aspects that shape modern CR. By addressing the interplay between emerging digital interventions and conventional rehabilitation models, it underscores how technology can bridge gaps in accessibility and adherence. Despite these promising developments, challenges of equity, standardization, and global implementation remain. Future research should focus on developing unified guidelines, expanding longitudinal follow-up, and optimizing individualized rehabilitation pathways to ensure that CR continues to evolve as a patient-centered, accessible, and data-driven field.
